# Compound K, a metabolite of ginseng saponin, induces apoptosis via caspase-8-dependent pathway in HL-60 human leukemia cells

**DOI:** 10.1186/1471-2407-9-449

**Published:** 2009-12-18

**Authors:** Sung-Hee Cho, Kyung-Sook Chung, Jung-Hye Choi, Dong-Hyun Kim, Kyung-Tae Lee

**Affiliations:** 1 Department of Pharmaceutical Biochemistry, College of Pharmacy, Kyung-Hee University, Seoul 130-701, South Korea; 2 Department of Oriental Pharmaceutical Science, College of Pharmacy, Kyung-Hee University, Seoul 130-701, South Korea; 3 Department of Biomedical Science, College of Medical Science, Kyung-Hee University, Seoul 130-701, South Korea

## Abstract

**Background:**

Compound K [20-*O*-β-(D-glucopyranosyl)-20(S)-protopanaxadiol], a metabolite of the protopanaxadiol-type saponins of *Panax ginseng *C.A. Meyer, has been reported to possess anti-tumor properties to inhibit angiogenesis and to induce tumor apoptosis. In the present study, we investigated the effect of Compound K on apoptosis and explored the underlying mechanisms involved in HL-60 human leukemia cells.

**Methods:**

We examined the effect of Compound K on the viabilities of various cancer cell lines using MTT assays. DAPI assay, Annexin V and PI double staining, Western blot assay and immunoprecipitation were used to determine the effect of Compound K on the induction of apoptosis.

**Results:**

Compound K was found to inhibit the viability of HL-60 cells in a dose- and time-dependent manner with an IC_50 _of 14 μM. Moreover, this cell death had typical features of apoptosis, that is, DNA fragmentation, DNA ladder formation, and the externalization of Annexin V targeted phosphatidylserine residues in HL-60 cells. In addition, compound-K induced a series of intracellular events associated with both the mitochondrial- and death receptor-dependent apoptotic pathways, namely, (1) the activation of caspases-3, -8, and -9; (2) the loss of mitochondrial membrane potential; (3) the release of cytochrome *c *and Smac/DIABLO to the cytosol; (4) the translocation of Bid and Bax to mitochondria; and (5) the downregulations of Bcl-2 and Bcl-xL. Furthermore, a caspase-8 inhibitor completely abolished caspase-3 activation, Bid cleavage, and subsequent DNA fragmentation by Compound K. Interestingly, the activation of caspase-3 and -8 and DNA fragmentation were significantly prevented in the presence of cycloheximide, suggesting that Compound K-induced apoptosis is dependent on *de novo *protein synthesis.

**Conclusions:**

The results indicate that caspase-8 plays a key role in Compound K-stimulated apoptosis via the activation of caspase-3 directly or indirectly through Bid cleavage, cytochrome *c *release, and caspase-9 activation.

## Background

**Figure 1:**
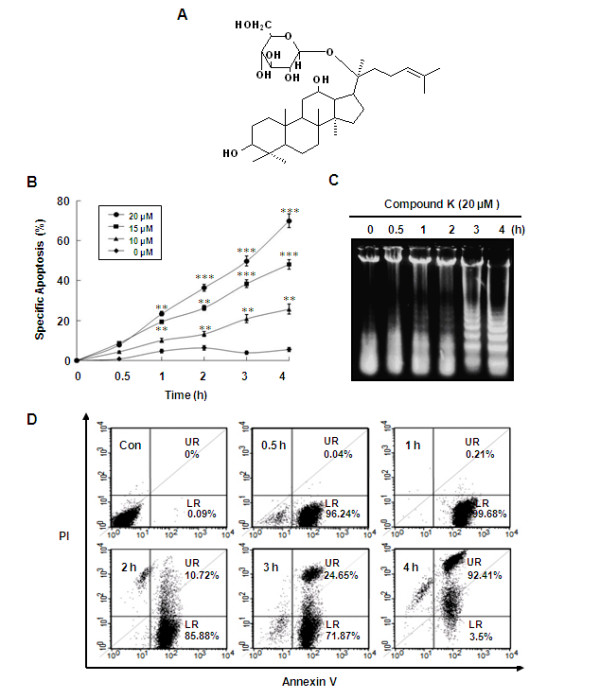
**Compound K induced apoptosis in HL-60 cells**. (A) The chemical structure of Compound K. (B) Cells were treated with various concentrations (10, 15, 20 μM) of Compound K for the indicated times. Extents (%) of DNA fragmentation were determined by fluorometric method using DAPI, as described in Methods. Data are presented as means ± SD of three independent experiments. *P < 0.05, **P < 0.01 and ***P < 0.001 vs. the control group; the significances of differences between treatments were determined using the Student's *t*-test. (C) Cells were treated with or without 20 μM Compound K for the indicated times. Fragmentation of genomic DNA was extracted and resolved on 2% agarose gels. Apoptotic DNA fragmentation was visualized by ethidium bromide staining. (D) Cells treated with or without 20 μM of Compound K for the indicated times were co-stained with PI and FITC-conjugated Annexin V, which specifically detected the translocation of phosphatidylserine (PS). Cells were then examined by flow cytometry (LR; low right, UR; upper right).

Ginseng, the root and rhizomes of different *Panax *species (Araliaceae), is one of the most commonly used as traditional medicines in East Asia. Furthermore, the saponins of ginseng (ginsenosides) are its major active components and have been shown to possess anti-inflammatory, anti-tumor, and neuroprotective activities [1]. The pharmacological actions of these ginsenosides have been attributed to their biotransformations by intestinal bacteria [2]. Protopanaxadiol ginsenoside is metabolized primarily to 20-*O*-β-(D-glucopyranosyl)-20(S)-protopanaxadiol (Compound K, Figure 1A) by intestinal bacteria via the stepwise cleavage of sugar moieties [3]. Furthermore, Compound K has been shown to inhibit glucose uptake and to reverse multi-drug resistance in tumor cells, but to be non-toxic to normal cells [4]. Compound K has also been reported to reverse benzo [a]pyrene-induced mutagenicity nd clastogenic activity [5], to inhibit tumor metastasis by suppressing invasion [6], and to stimulate apoptosis in several tumor cell lines [7,8].

Apoptosis is a selective process of physiological cell deletion that plays an important role in the balance between cellular replication and death. Furthermore, it has been suggested that some cancer chemotherapeutics and chemopreventives exert their effects by triggering either apoptotic cell death or cell cycle transition, and accordingly, the induction of tumor cell apoptosis is used to predict tumor treatment response [9,10]. Apoptotic signaling can proceed via two pathways, i.e., via death receptors expressed on the plasma membranes of cells or alternatively via mitochondria, which contain several proteins that regulate apoptosis. The death receptor pathway is initiated by the ligation of membrane bound tumor necrosis factor (TNF) or Fas receptors, which result in a caspase-8-dependent cascade and subsequent cell death [11]. During this cascade, caspase-8 cleaves Bid and induces cytochrome *c *release and/or directly activates caspase-3 [12]. On the other hand, the mitochondrial pathway involves cytochrome *c *release, which leads to caspase-9 activation and a proteolytic caspase cascade [13]. Thus, caspase cascades appear to be a central component in the apoptotic process. However, accumulating evidence indicates that apoptosis is also induced by caspase-independent pathways [14], which has been suggested to involve mitochondrial release of apoptosis-inducing factor (AIF) and/or endonuclease G [15,16].

Human leukemia results from multiple mutations that lead to abnormalities in the expressions and functions of genes that maintain the delicate balance between proliferation, differentiation, and apoptosis. Continued research on the molecular aspects of leukemia cells has resulted in the developments of several potentially useful therapeutic agents. In this context, we investigated that Compound K induced apoptosis and the mechanism involved the activation of caspase-8 system.

## Methods

### Cells and reagents

HL-60 human promyelocytic leukemia, U-937 human histocytic lymphoma, HeLa human negroid cervix epitheloid carcinoma, A549 human lung adenocarcinoma, HepG2 human hepatoblastoma, P388 mouse lymphoblast, and A431 human epidermoid carcinoma cells were obtained from the Korean cell line bank (KCLB, Seoul, Korea) and were cultured according to the KCLB recommendations.

Compound K used in this study was isolated from *Panax ginseng *and structural identities were determined spectroscopically (^1^H and ^13^NMR, IR, MS) as described previously [17]. The identity of isolated compound was confirmed by LC-MS and was found to be >98% pure. RPMI 1640 medium, fetal bovine serum (FBS), penicillin, and streptomycin were obtained from Life Technologies Inc. (Grand Island, NY). 3-(4,5-Dimethylthiazol-2-yl)-2,5-diphenyl-tertazolium bromide (MTT), dimethyl sulfoxide (DMSO), RNase A, leupeptin, aprotinin, phenylmethylsulfonylfluoride (PMSF), 4',6-diamidino-2-phenylindole-dihydrochloride (DAPI), Triton X-100, Nonidet P-40, protein A/G-Sepharose beads, and propidium iodide (PI) were purchased from Sigma Chemical Co. (St. Louis, MO). The following antibodies for caspase-3, poly(ADP-ribose) polymerase (PARP), Bid, tBid, Bax, Bcl-2, Bcl-xL, anti-Smac/DIABLO, Fas, FasL, α-tubulin and β-actin were purchased from Santa Cruz Biotechnology (Santa Cruz, CA). The antibodies for Fas-associated death domain protein (FADD), X-linked inhibitor of apoptosis protein (XIAP), caspase-8, caspsae-9, and cytochrome *c *were purchased from BD Biosciences, Pharmingen (San Diego, CA). The antibody for COX IV was purchased from Cell Signaling Technology (Beverly, MA), and Bax 6A7 antibody was obtained from Trevigen, Inc. (Gaithersburg, MD). z-VAD-fmk and z-DEVD-fmk, z-IETD-fmk, and z-LEHD-fmk were purchased from Calbiochem (Bad Soden, Germany).

### MTT assay

The cytotoxicity was assessed using a MTT assay [18]. Briefly, the cells (5 × 10^4^) were seeded in each well containing 100 μL of the RPMI medium supplemented with 3% FBS in a 96-well plate. After 24 h, various concentrations of Compound K were added. After 48 h, 50 μL of MTT (5 mg/mL stock solution) was added and the plates were incubated for an additional 4 h. The medium was discarded and the formazan blue, which was formed in the cells, was dissolved with 100 μLDMSO. The optical density was measured at 540 nm.

### Detection and quantification of DNA fragmentation

DNA fragmentation was quantified using DAPI staining and analysis of DNA fragmentation by agarose gel electrophoresis was performed as described previously [19]. In brief, cells were lysed in a solution of 5 mM Tris-HCl (pH 7.4) and 1 mM EDTA containing 0.5% (w/v) Triton X-100 for 20 min on ice. After centrifugation at 25,000 *g *for 20 min, the lysate and supernatant were sonicated for 15 sec and the level of DNA in each fraction was measured by a fluorometric method using DAPI. The amount of the fragmented DNA was calculated as the ratio of the amount of DNA in the supernatant to that in the lysate. Genomic DNA was prepared and was performed in a 1.5% (w/v) agarose gel in 40 mM Tris-acetate buffer (pH 7.4) at 50 V for 1 h. The fragmented DNA was visualized by staining with ethidium bromide after electrophoresis.

### PI and Annexin V double staining

For PI and Annexin V double staining, cells were suspended with 100 μl of binding buffer (10 mM HEPES/NaOH, 140 mM NaCl, 2.5 mM CaCl_2_, pH 7.4) and stained with 5 μL of FITC-conjugated Annexin V and 10 μL of PI (50 μg/mL) for 15 min at room temperature in dark place and then added 400 μl binding buffer, and analyzed by the fluorescence-activated cell sorting (FACS) cater-plus flow cytometry (Becton Dickinson Co, Heidelberg, Germany)

### Analysis of mitochondrial membrane potential (MMP, ΔΨ_*m*_)

Changes in mitochondrial transmembrane potential were monitored by flow cytometric analysis. Cells were incubated with 50 nM 3,3'-dihexyloxacarbocyanine iodide (DiOC_6_) for 30 min, washed twice with PBS, and analyzed by flow cytometric analysis (Becton Dickinson Co, Heidelberg, Germany) with excitation and emission settings of 484 and 500 nm, respectively. To ensure that DiOC_6 _uptake was specific for *ΔΨ*_*m*_, we also treated cells with 50 μM carbonyl cyanide *m*-chlorophenylhydrazone (CCCP) or 5 μM cyclosporine A (CsA). CCCP was used as a reference depolarizing agent and CsA was used as an inhibitor of mitochondrial permeability transition.

### Protein extraction and Western blot analysis

Cells were collected by centrifugation at 200 *g *for 10 min at 4°C. The cells were then washed twice with ice-cold PBS, and centrifuged at 200 *g *for 5 min. The cell pellet obtained was then resuspended in 1× protein lysis buffer (Intron, Seoul, Korea). Equal amounts of cell lysates were separated by SDS- polyacrylamide gel and transferred to nitrocellulose membranes for Western blot analysis using the indicated primary antibodies. HRP-conjugated secondary antibodies were detected using an ECL (Amersham, Buckingham-shire, England) detection system.

### Preparation of mitochondrial proteins

Cells were collected by centrifugation at 200 *g *for 10 min at 4°C. The cells were then washed twice with ice-cold PBS, and centrifuged at 200 *g *for 5 min. The cell pellet obtained was then resuspended in ice-cold cell extraction buffer (20 mM HEPES-KOH, pH 7.5, 10 mM KCl, 1.5 mM MgCl_2_, 1 mM EDTA, 1 mM EGTA, 1 mM dithiothreitol, 100 μM PMSF, and protease inhibitor cocktail) for 30 min on ice. The cells were then homogenized with a glass dounce and a B-type pestle (80 strokes), homogenates were spun at 15,000 *g *for 15 min at 4°C, and the supernatant (cytosolic fraction) was removed whilst taking care to avoid the pellet. The resulting pellet (mitochondrial fraction) was resuspended in extraction buffer. Cell lysates were fractionated in SDS- polyacrylamide gels and transferred to nitrocellulose membranes fot immunoblot analysis using the indicated primary antibodies. Immuno-positive bands were visualized by ECL kit (Amersham, Buckingham-shire, England).

### Immunoprecipitation

After harvesting and washing, pellets were lysed in EBC buffer (30 mM Tris-HCl, pH 7.5, 150 mM NaCl, 10% glycerol, 1 mM EDTA, 2.5 mM EGTA, 5 mM NaF, 0.1 mM Na_3_VO_4_, 1% Triton X-100 and protease inhibitor) for 15 min on ice. After centrifugation (10,000 *g*, 5 min), protein concentrations were determined. Equal amount of protein (100 μg) was incubated with anti-Smac/DIABLO, anti-tBid, anti-Bax, and anti-Fas antibodies for 12 h at 4°C, followed by incubation with 40 μL protein A-Sepharose beads for 4 h. The protein complex was washed 4 times with EBC buffer and released from the beads by boiling in 6× sample buffer (350 mM Tris, pH 6.8, 10% SDS, 30% β-mercaptoethanol, 6% glycerol, 0.12% bromophenolblue) for 5 min. The reaction mixture was then resolved by a 10 - 12% SDS- polyacrylamide gels, transferred to nitrocellulose membrane and probed with anti-XIAP, anti-Bax 6A7 (Trevigen Inc., Gaithersburg, MD), anti-Bcl-xL, anti-Bcl-2, anti-tBid, anti-Bax, anti-caspase-8, anti-Fas, anti-FasL, and anti-FADD antibodies. Immuno-positive bands were visualized by ECL kit (Amersham, Buckingham-shire, England).

### Statistical analysis

Data presented are the means ± S.D. of results from three independent experiments with similar patterns. *P < 0.05, ** *P *< 0.01, *** *P *< 0.001 vs control group, ^†^*P *< 0.05, ^†† ^*P *< 0.01, ^††† ^*P *< 0.001 *vs *Compound K-treated group; significance of difference between treated groups by Student's *t*-test.

## Results

### Compound K inhibited HL-60 cell growth and induced apoptosis

We examined the effect of Compound K on the viabilities of various cancer cell lines using MTT assays, and assessed its effects using IC_50 _values (Table 1). IC_50 _values were found to fall in the range 14.1 - 59.4 μM, and Compound K was found to have marked cytotoxic effects on human leukemia cell lines, including HL-60 and U937. Further experiments were performed using HL-60 cells to evaluate the effect of Compound K on apoptosis and to identify the mechanism involved. To determine whether the cell growth inhibitory effect of Compound K is associated with the induction of apoptosis, DNA fragmentation was evaluated using DAPI assay and by agarose gel electrophoresis. DAPI assay revealed that Compound K caused DNA fragmentation, in a time- and dose-dependent manner in HL-60 cells (Figure 1B). Furthermore, a typical ladder pattern of internucleosomal DNA fragmentation was observed after treating HL-60 cells with Compound K (20 μM) for 4 h (Figure 1C). To further characterize Compound K-induced apoptosis, we assessed the translocation of phosphatidylserine (PS) using Annexin V and PI double staining. As shown in Figure 1D, cell numbers in lower-right quadrants, which correspond to early apoptotic cells (Annexin V-positive), were increased up to 99% after treating cells with Compound K (20 μM, for 1 h), and about 92% of cells were in the late apoptotic stage after 4 h.

**Table 1:** Cell viabilities of Compound K in various cancer cell lines *in vitro*

Cell lines	IC_50 _(μM)^a^
**HL-60**	14.1
**U937**	16.5
**HeLa**	29.9
**A549**	37.9
**HepG2**	59.4
**P388**	18.4
**A431**	37.9

^a^IC_50 _is defined as the concentration that results in a 50% decrease in the number of cells compared to that of the control cultures in the absence of Compound K. The values represent the means of results from three independent experiments with similar patterns.

### Caspase involvement in Compound K-induced apoptosis

**Figure 2:**
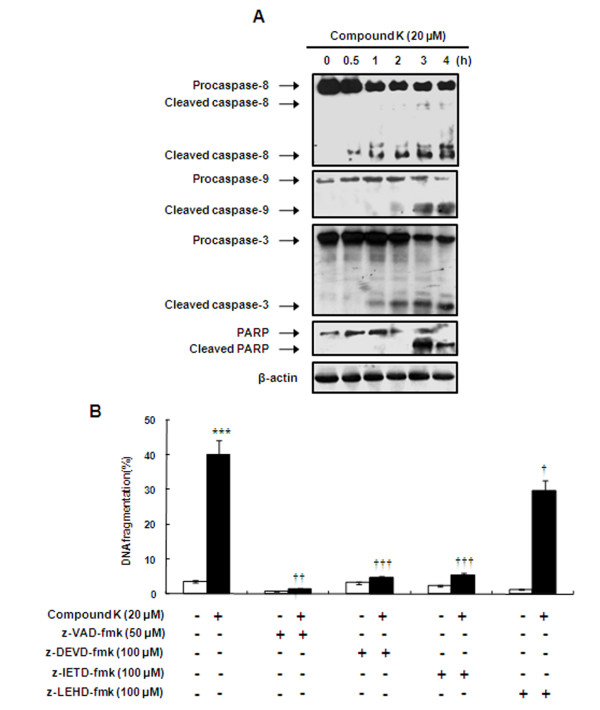
**Compound K induced the apoptosis of HL-60 cells via caspase activation**. (A) Cells were treated with 20 μM of Compound K for the indicated times. Caspase-8, caspase-9, caspase-3, and PARP were analyzed by Western blotting. β-actin was used as an internal control. Experiments were repeated three times and similar results were obtained. (B) Effects of the caspase inhibitors (z-VAD-fmk, z-DEVD-fmk, z-IETD-fmk and z-LEHD-fmk) on apoptosis were determined by testing for Compound K-induced DNA fragmentation. HL-60 cells were pretreated with/without 50 μM z-VAD-fmk, 100 μM z-DEVD-fmk, 100 μM z-IETD-fmk or 100 μM z-LEHD-fmk for 1 h, and then treated with Compound K (20 μM for 2 h). DNA fragmentation was measured by DAPI staining. White square: None, Black square: Compound K. The data presented are the means ± SD of results obtained from three independent experiments. ***P < 0.001 *vs*. control group, ^††^*P *< 0.01, ^†††^*P *< 0.001 *vs*. the Compound K-treated group; significances were determined using the Student's *t*-test.

To identify the mechanism involved in Compound K-induced apoptosis, we investigated the activation of caspase-8, -9, -3, and the cleavage of PARP. As shown in Figure 2A, Compound K significantly increased the activation of caspase-8, -9, -3 and the cleavage of PARP (an endogenous substrate of caspase-3) in a time-dependent manner. Following treatment with Compound K, the cleaved form **s **of caspase-8 and -3 were observed at 0.5 h and 1 h, respectively, whereas caspase-9 and PARP were cleaved after 3 h of treatment in HL-60 cells. Additionally Figure 2B showed that z-VAD-fmk (a broad caspase inhibitor), z-DEVD-fmk (a specific caspase-3 inhibitor), and z-IETD-fmk (a specific caspase-8 inhibitor) all markedly attenuated Compound K-stimulated DNA fragmentation, thus indicating that Compound K-induced apoptosis is largely dependent on the activation of caspases-8 and -3. In contrast, z-LEHD-fmk (a specific caspase-9 inhibitor) only partially inhibited DNA fragmentation.

### Compound K induced apoptosis involving the loss of ΔΨ_*m *_and the release of cytochrome c and Smac/DIABLO

**Figure 3:**
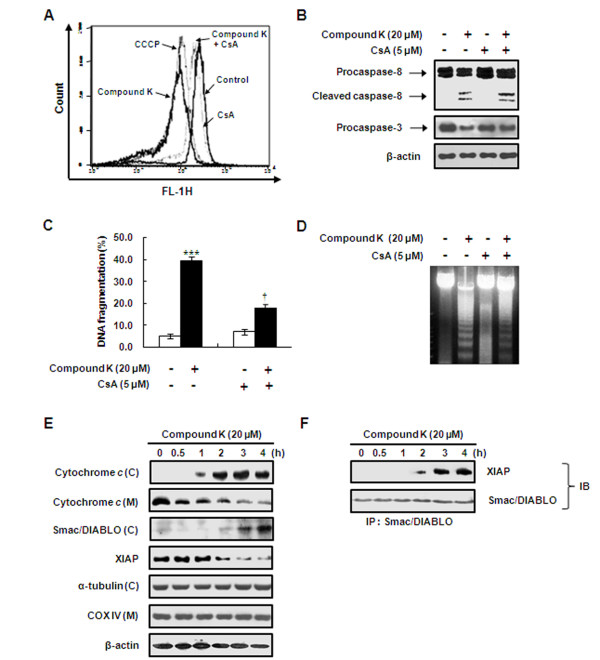
**Compound K caused a loss in mitochondrial membrane potential and induced the translocations of cytochrome *c *and Smac/DIABLO from mitochondria to the cytoplasm in HL-60 cells**. (A) Cells were pretreated with 5 μM cyclosporin A (CsA) for 30 min and then treated with 20 μM of Compound K for 2 h, stained with DiOC_6_, and analyzed by flow cytometry. CCCP (100 μM) was used as a positive control. (B) Cells were pretreated as described in the legend of Figure 3 (A). Cleavages of procaspase-8 and -3 were analyzed by Western blotting. DNA fragmentation was measured using (C) DAPI assays and (D) fragmented genomic DNA was extracted and resolved on 2% agarose gel. Data presented are the means ± SD of three independent experiments. ***P < 0.001 *vs*. untreated controls, ^†^*P *< 0.05 vs. Compound K-treated cells; significances were determined using the Student's *t*-test. (E) Cells were harvested after being incubated with Compound K at 20 μM for the indicated times. Mitochondrial (M) and cytosolic (C) fractions were prepared as described in Methods. Cytochrome *c*, Smac/DIABLO, and XIAP were analyzed by Western blotting. α-tubulin, cytochrome *c *oxidase (COX) IV, and β-actin were used as internal controls. (F) Compound K induced an interaction between XIAP and Smac/DIABLO. Cells were treated with 20 μM of Compound K for the indicated times. Smac/DIABLO was immunoprecipitated from total protein lysates and proteins were subjected to Western blotting for anti-XIAP antibody (IP: immunoprecipitation, IB; immunoblotting).

Because Compound K induced caspase-9 cleavage in HL-60 cells, the initial caspase in the mitochondrial apoptotic pathway, we investigated whether Compound K was capable of inducing *ΔΨ*_*m *_depolarization using DiOC_6_, a mitochondria-specific voltage-dependent dye. Treatment of cells with Compound K (20 μM) caused the dissipation of *ΔΨ*_*m*_, as did CCCP (the positive control) (Figure 3A). In order to determine whether this loss of *ΔΨ*_*m *_was associated with Compound K-induced apoptosis, we utilized CsA (5 μM; an inhibitor of mitochondrial permeability transition). Pretreatment with CsA significantly inhibited the Compound K-induced loss of *ΔΨ*_*m *_and caspase-3 activation, but had no effect on caspase-8 activation (Figure 3B). Additionally, DAPI staining and agarose gel electrophoresis assays showed that CsA partially inhibited Compound K-induced DNA fragmentation (Figure 3C and 3D), indicating that *ΔΨ*_*m *_is partially involved in Compound K-induced apoptosis. Furthermore the levels of cytosolic cytochrome *c *and Smac/DIABLO were elevated by Compound K in HL-60 cells, whereas X-linked IAP (XIAP) levels decreased, which suggests the involvement of the mitochondrial pathway (Figure 3E). To determine whether Smac/DIABLO release by Compound K is able to dissociate XIAP from caspases, we immunoprecipitated Smac/DIABLO, and detected bound XIAP by Western blotting. We found that Compound K significantly increased the interaction between Smac/DIABLO and XIAP (Figure 3F). The above results suggest that Compound K stimulates the release of Smac/DIABLO from mitochondria and the association between Smac/DIABLO and XIAP, and that this inhibits the XIAP - caspase interaction and consequently stimulates apoptosis.

### Compound K induced the translocations of tBid and Bax and downregulated Bcl-2 and Bcl-xL

**Figure 4:**
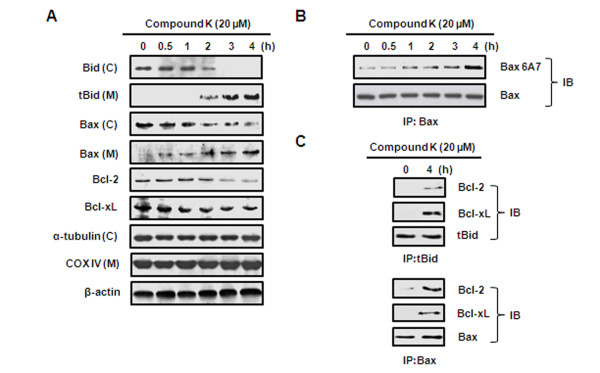
**Effect of Compound K on the expressions of Bcl-2 family proteins for Compound K-induced apoptosis in HL-60 cells**. (A) Cells were harvested after being incubated with Compound K (20 μM) for the indicated times. Mitochondrial (M) and cytosolic (C) fractions were prepared as described in Methods. Bid, tBid, Bax, Bcl-2, and Bcl-xL were analyzed by Western blotting. α-tubulin, COX IV and β-actin were used as internal controls. (B) HL-60 cells were treated with 20 μM of Compound K for 4 h and cell lysates were prepared. Bax was immunoprecipitated from total protein lysates and proteins were subjected to Western blotting for anti-Bax 6A7 using specific antibody. (C) Compound K-induced interactions between Bcl-2 family members. tBid and Bax were immunoprecipitated using specific antibodies from total protein lysates and Western blotting was conducted for Bcl-2 and Bcl-xL (IP; immunoprecipitation, IB; immunoblotting).

To investigate the mechanism underlying Compound K-induced *ΔΨ*_*m *_changes in HL-60 cells, we examined the translocations of cytosolic Bid and Bax into mitochondria after treating cells with Compound K. As shown in Figure 4A, treatment with Compound K reduced the cytosolic levels of pro-apoptotic Bid and Bax, but increased their mitochondrial levels. In addition, the mitochondrial expressions of Bcl-2 and Bcl-xL (mitochondrial anti-apoptotic proteins) were reduced by Compound K. It has been shown that apoptotic stimuli trigger a conformational change in Bax and induce its translocation to mitochondria [20]. As shown in Figure 4B, immunoprecipitation assays were performed using an anti-Bax 6A7 antibody recognizing only conformationally changed Bax, and Compound K induced a time-dependent increase in conformationally changed Bax. Furthermore, treatment with Compound K for 4 h, significantly enhanced bindings of tBid to Bcl-2 or Bcl-xL, and increased association between Bax and Bcl-2 or Bcl-xL (Figure 4C). These results suggest the Compound K-induced translocations of tBid and Bax stimulates their direct binding to Bcl-2 or Bcl-xL in mitochondria, and that this perturbs the balance between anti- and pro-apoptotic Bcl-2 family proteins in mitochondria.

### Compound K induced apoptosis via the caspase-8- dependent pathway

**Figure 5:**
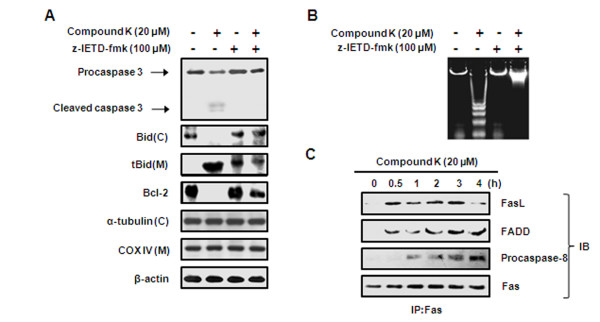
**Effect of Compound K on the requirement for caspase-8 for Compound K-induced apoptosis in HL-60 cells**. (A) HL-60 cells were pretreated with/without 100 μM z-IETD-fmk (a specific caspase-8 inhibitor) for 1 h and then treated with 20 μM of Compound K for 4 h. Mitochondrial (M) and cytosolic (C) fractions were prepared as described in Methods. Caspase-3, Bid, and tBid were analyzed by Western blotting. Anti-α-tubulin, anti-COX IV and anti-β-actin were used as internal controls. (B) Samples were treated as described in the legend of Figure 5(A). Genomic DNA fragments were extracted and resolved on 2% agarose gel. Apoptotic DNA fragmentation was visualized by ethidium bromide staining. (C) Fas was immunoprecipitated from total protein lysates and proteins were subjected to Western blotting for anti-FasL, anti-FADD, or anti-caspase-8 antibodies (IP: immunoprecipitation, IB; immunoblotting).

Because Compound K induced caspase-8 activation and Bid cleavage (Figure 2A and 4A), we further examined the mechanism of caspase-8 activation during Compound K-induced apoptosis using z-IETD-fmk. As shown in Figure 5A, treatment with z-IETD-fmk inhibited caspase-3 activation, Bid translocation, and Bcl-2 downregulation by Compound K. Furthermore, Compound K-induced internucleosomal DNA fragmentation was markedly abolished in the presence of z-IETD-fmk (Figure 5B). To determine whether DISC (death-inducing signaling complex) formation is involved in the Compound K-induced activation of caspase-8 and apoptosis, Fas was immunoprecipitated and bindings of FasL, FADD, and procaspase-8 to Fas were evaluated by Western blotting (Figure 5C). The association of Fas with FasL and FADD was observed from 0.5 h after Compound K treatment, while the binding of procaspase-8 to Fas was observed at 1 h. These findings suggest that Compound K stimulates DISC formation and the subsequent activation of procaspase-8.

### Cycloheximide prevented Compound K-induced caspase-8 and -3 activation and DNA fragmentation

**Figure 6:**
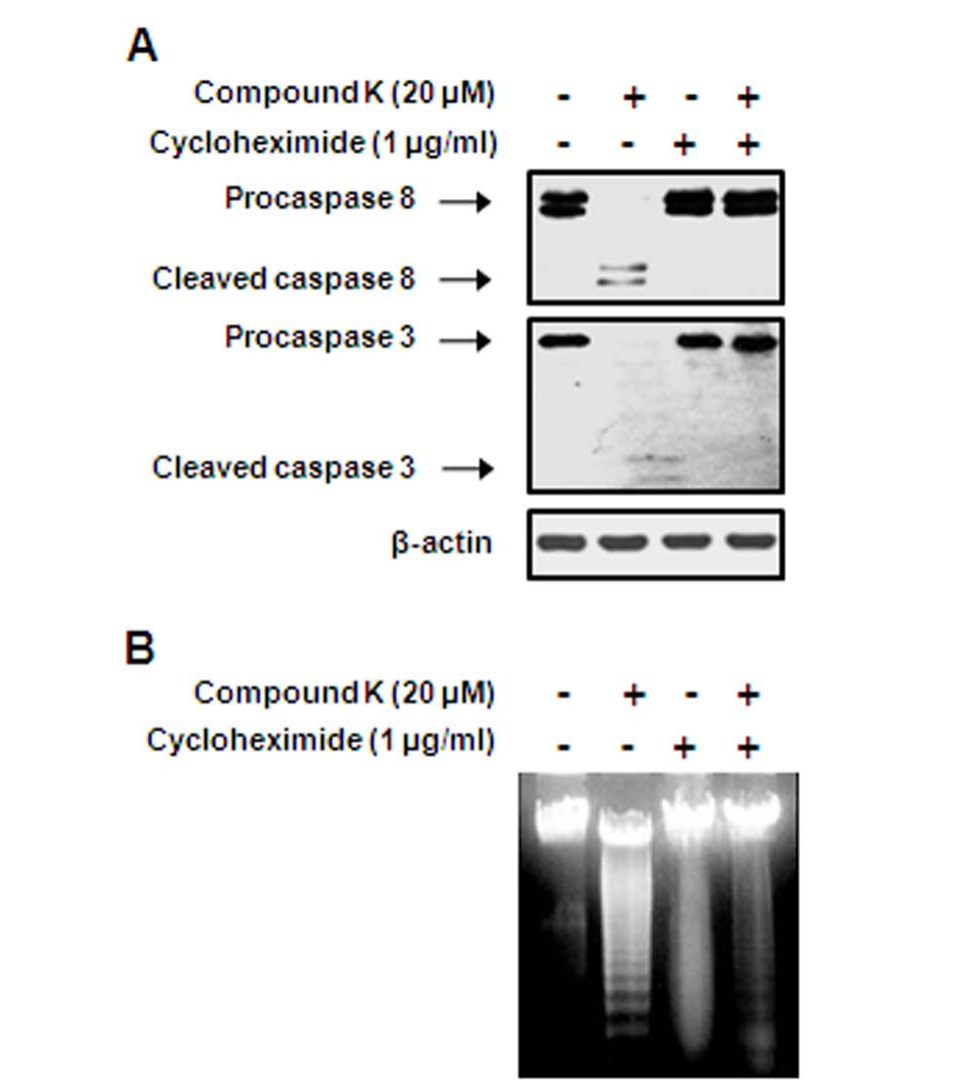
**Effect of cycloheximide on Compound K-induced caspase activities and DNA fragmentation in HL-60 cells**. (A) Cells were pretreated with 1 μg/mL cycloheximide for 1 h and then treated with 20 μM of Compound K for 4 h. Cleavages of procaspase-8 and -3 were analyzed by Western blotting. (B) Compound K-induced DNA fragmentation was visualized by ethidium bromide staining.

To test the possibility that new protein synthesis is required for Compound K-induced apoptosis, HL-60 cells were preincubated with cycloheximide (1 μg/mL for 1 h); a protein synthesis inhibitor and then treated with Compound K for 4 h. Cycloheximide completely inhibited the activation of caspase-8 and -3 and DNA fragmentation (Figures 6A and 6B) by Compound K, indicating that *de novo *protein synthesis is required for Compound K-induced apoptosis.

## Discussion

Compound K is a novel ginseng saponin metabolite that is formed by the action of intestinal bacteria on ginseng extract in man and rats [3]. The intestinal bacteria *Prevotella oris*, which is responsible for the hydrolysis of ginsenoside Rb1 to Compound K, has been found in 79% of human fecal specimens [21]. Since Compound K was detected in blood after oral administration of ginsenoside Rb1 to mice, it has been speculated that it is probably the major form of protopanaxadiol saponin absorbed by the intestine. Furthermore, evidence that Compound K is the active metabolite responsible for the anti-carcinogenic effects of ginseng saponins has prompted several groups to investigate its effects in detail [22-26].

Compound K has been reported to have potent anti-tumorigenic activity, and in particular, to inhibit 12-O-tetradecanoylphorbol-13-acetate (TPA)-induced tumor promotion in mouse skin, and COX-2 expression in mouse skin and in cultured human mammary epithelial cells [22]. In addition, Compound K has been reported to inhibit glucose uptake by tumor cells and to reverse multidrug resistance in bacterial and tumor cells [23]. Compound K was also found to have antiangiogenic activities in a spontaneous metastasis model [24,27], and to inhibit the expression of matrix metalloproteinase (MMP)-9, which regulates the growth and invasiveness of brain tumors [24]. It has also been reported to induce apoptosis in activated rat hepatic stellate [25] and hepatoma [7,26] cancer cells. However, the effects of Compound K on leukemia have not been studied in detail, although Lee *et al *found that Compound K induced apoptosis via the cytochrome *c*-mediated activation of caspase-3 in HL-60 cells [28]. Our present results confirm those of Lee et al., in as much as Compound K showed cytotoxic and apoptosis-inducing activities in HL-60 cells.

Apoptosis is a fundamental cellular activity and provides protection against cancer development by eliminating genetically altered cells and hyper-proliferative cells. Thus, defects in apoptosis signaling pathways contribute to carcinogenesis and chemoresistance [29]. As mentioned above, there are two major apoptotic pathways; the extrinsic death receptor-mediated pathway and the intrinsic mitochondria-mediated pathway, and truncated Bid protein provides cross-talk between the two [30]. Both of these pathways are regulated by caspases, which are responsible, either directly or indirectly, for the cleavages of cellular proteins, a characteristic of apoptosis [31]. In the present study, Compound K increased the levels of cleaved caspase-8, -9, and -3 and PARP in a time-dependent manner and pretreatment with various caspase inhibitors markedly prevented Compound K-induced DNA fragmentation (Figure 2). These findings indicate that caspase-3 and -8 play fundamental roles in Compound K-induced apoptosis in HL-60 cells. It is noteworthy that the specific inhibition of caspase-9 by z-LEHD-fmk had only a mild inhibitory effect on Compound K-stimulated apoptosis. In this regard, it appears that activation of the intrinsic mitochondria-mediated pathway alone is not sufficient to explain the apoptotic effects of Compound K in HL-60 leukemia cells. In addition, caspase-9 was activated about 2 h after treatment with Compound-K while activation of caspase-3 was observed at 0.5 h. Recent studies have suggested that caspase-3 has feedback action on caspase-9 [32]. For example, caspase-3-mediated activation of caspase-9 was involved in cisplatin-induced apoptosis [33]. This data suggested a possibility that tBid/caspase 9 activation is a consequence of active caspase 3 in the Compound-K-induced apoptosis.

Activation of the intrinsic mitochondrial pathway leads to the release of Smac/DIABLO, which removes the IAP blockage of caspase activation [34]. The IAP family, which includes XIAP and survivin, functions by binding to and inhibiting several caspases [35]. In the present study, we observed Smac/DIABLO increased in cytoplasm and correspondingly decreased in mitochondria after treating cells with Compound K (data not shown), which suggests that the translocation of Smac/DIABLO from mitochondria to cytoplasm contributes to Compound K-induced apoptosis in HL-60 leukemia cells. Furthermore, Compound K-induced releases of cytochrome *c *and Smac/DIABLO from mitochondria were seemingly associated with mitochondrial depolarization (Figure 3A). Bid, a BH3-only proapoptotic member of the Bcl-2 family, undergoes proteolysis by caspase-8 activated by cell surface death receptors, such as, Fas or TNF [36,37], and the tBid so produced, translocates to mitochondria and binds to anti-apoptotic proteins, such as, Bcl-2 and Bcl-xL, which leads to a conformational change in Bax, mitochondrial depolarization, and cytochrome *c *release from mitochondria [38]. Moreover, conformationally altered Bax stimulates the release of Smac/DIABLO from mitochondria [34], and the cytochrome *c*-releasing activity of Bid by cell surface death receptors such as Fas and TNF is antagonized by Bcl-2 [37].

In the present study, Compound K-induced releases of cytochrome *c *and Smac/DIABLO from mitochondria were seemingly associated with mitochondrial depolarization (Figure 3) and Compound K increased the translocations of Bid and Bax to mitochondria and their bindings to Bcl-2 and Bcl-xL, which is presumed to induce a conformational change in Bax and to reduce the mitochondrial levels of Bcl-2 and Bcl-xL in HL-60 cells (Figure 4). These findings suggest that the tBid triggered-imbalance between pro- and anti-apoptotic Bcl-2 family members is associated with the mitochondrial membrane depolarization that leads to the releases of cytochrome *c *and Smac/DIABLO in Compound K-treated cells.

The bindings of FasL, TRAIL, and TNF to their cell surface receptors are known to lead to caspase-8 activation, which then amplifies the apoptotic signal either by directly activating downstream caspases or by cleaving Bid, a BH3-only proapoptotic member of the Bcl-2 family [30]. In the present study, caspase-8 inhibition prevented the cleavages of Bid and caspase-3 and subsequent apoptosis (Figure 5A), which suggests that Bid is a substrate of caspase-8, and that caspase-8 is required for Compound K-induced apoptosis. Furthermore, we found that Compound K stimulates DISC formation with the FasL, Fas, FADD, and procaspase-8, which indicates that Compound K-induced caspase-8 activation is probably due to the stimulation of the death receptors. The mechanism by which Compound K promotes DISC formation appears to be dependent on *de novo *protein synthesis since the Compound K-induced activation of caspases-8 and -3 and DNA fragmentation were prevented by cycloheximide. During our earlier studies, we found FasL is abundantly expressed in HL-60 cells, and that Compound K increases Fas protein expression in a time-dependent manner, but does not affect FasL (data not shown). In this regard, we speculate that Compound K stimulates apoptosis by upregulating Fas in HL-60 cells. Indeed, in a previous study, the triterpene saponin platycodin D was demonstrated to induce DNA fragmentation and caspase-3/-8 activation within 3 h in immortalized keratinocyte HaCa cells [39]. Recent studied have demonstrated that the general translation rate is about 2-2.8 amino acid/sec suggestting that it would take only 15 min to make a 50% change in protein concentration [40,41]. Furthermore, Fas and FasL protein upregulations were observed at 15 and 30 min, respectively, after platycodin D treatment, and NF-κB activation (mediated by the degradation of IκB) was found to be involved in these upregulations. Other studies have also demonstrated the involvement of NF-κB in the regulation of Fas and FasL expression in a variety of cell lines [42,43]. However, whether Compound K-induced apoptosis is mediated by NF-κB-regulated Fas expression in HL-60 cells remains to be determined. Taken together, we hypothesize Compound K-induced apoptosis in HL-60 cells is associated with the upregulations of cleaved caspases-8 and -3, tBid, and mitochondrial membrane depolarization, and that crosstalk between the caspase-8/Bid and mitochondrial pathways appears to contribute to Compound K-induced apoptosis.

## Conclusion

Our results demonstrate that Compound K inhibit the viability of HL-60 cells and these effects were mediated through the induction of apoptosis. Compound K induced the activation of caspase-3, -8, and -9, and modulation of Bcl-2 families. In addition, a caspase-8 inhibitor completely abolished caspase-3 activation, Bid cleavage, and subsequent DNA fragmentation by Compound K. Therefore caspase-8 plays a key role in Compound K-stimulated apoptosis. Based on these finding, we suggest that Compound K may be used as a potential therapeutic and chemopreventive agent for leukemia via the potent apoptotic activity.

## Abbreviations

Caspase: cystein arspartyl-specific protease; Smac/DIABLO: second mitochondria-derived activator of caspase/direct inhibitor of apoptosis protein binding protein with low pI; XIAP: X-linked inhibitor of apoptosis protein; FADD: Fas-associated death domain protein.

## Competing interests

The authors declare that they have no competing interests.

## Authors' contributions

SHC and KSC performed major experimental work, conceived of the study in its design and coordination, and drafted the manuscript. DHK provided the Compound K in collaboration. JHC and KTL participated in the overall design of study and helped to draft the manuscript. All authors read and approved the final manuscript.

## Pre-publication history

The pre-publication history for this paper can be accessed here:

http://www.biomedcentral.com/1471-2407/9/449/prepub
